# Clinical performance of metagenomic next-generation sequencing for the rapid diagnosis of talaromycosis in HIV-infected patients

**DOI:** 10.3389/fcimb.2022.962441

**Published:** 2022-10-21

**Authors:** Yuhuan Mao, Hui Shen, Caili Yang, Qunying Jia, Jianying Li, Yong Chen, Jinwei Hu, Weiliang Huang

**Affiliations:** ^1^ Department of Laboratory Medicine, The First Hospital of Changsha, Changsha, China; ^2^ Hunan Key Laboratory of Oncotarget Gene and Clinical Laboratory, Hunan Cancer Hospital and the Affiliated Cancer Hospital of Xiangya School of Medicine, Central South University, Changsha, China

**Keywords:** mNGS, talaromycosis, *Talaromyces marneffei*, HIV/AIDS, diagnosis

## Abstract

**Background:**

Talaromycosis is an invasive endemic mycosis caused by the dimorphic fungus *Talaromyces marneffei* (*T. marneffei*, TM). It mainly affects immunodeficient patients, especially HIV-infected individuals, which causes significant morbidity and mortality. Culture-based diagnosis takes a long turnaround time with low sensitivity, leading to treatment delay. In this study, we aimed to evaluate the performance of Metagenomic Next-Generation Sequencing (mNGS) for the rapid diagnosis of talaromycosis in HIV-infected patients.

**Methods:**

Retrospectively analysis was conducted in HIV-infected cases at Changsha First Hospital (China) from January 2021 to March 2022. Patients who underwent routine microbiological examination and mNGS testing in parallel were enrolled. The clinical final diagnosis was used as a reference standard, and cases were classified into the TM group (60 cases) and the non-TM group (148 cases). The clinical performances of mNGS were compared with culture and serum Galactomannan (GM). The mixed infections detected by mNGS were analyzed. The impact of mNGS detection on treatment was also investigated.

**Results:**

The sensitivity of mNGS test reached 98.3% (95% CI, 89.8-99.9), which was significantly higher than culture (66.7% [95% CI, 53.2-77.9], *P* < 0.001) and serum GM (83.3% [95% CI, 71.0-91.2], *P* < 0.05). The specificity of 98.6% (95% CI, 94.7-99.7) was similar to culture (100.0% [95% CI, 96.8-100.0], *P* = 0.156), and superior to serum GM (91.9% [95% CI, 85.9-95.5], *P* < 0.05). In bronchoalveolar lavage fluid (BALF) samples, the positive rate of mNGS was 97.6%, which was significantly higher than culture (28.6%, *P <*0.001). mNGS has excellent performance in the identification of mixed infection in TM group patients. *Cytomegalovirus*, *Epstein-Barr virus* and *Pneumocystis jirovecii* were the most common concurrent pathogens. In summary, 60.0% (36/60) patients were added or adjusted to antimicrobial therapy after mNGS test.

**Conclusion:**

mNGS is a powerful technique with high specificity and sensitivity for the rapid diagnosis of talaromycosis. mNGS of BALF samples may be a good option for early identification of *T. marneffei* in HIV-infected individuals with manifestations of infection. Moreover, mNGS shows excellent performance in mixed infection, which benefits timely treatment and potential mortality reduction.

## 1 Introduction

*Talaromyces marneffei* (*Penicillium marneffei*) is a dimorphic pathogenic fungus, which is mainly epidemic in residents, returning travellers, and immigrants with immunocompromised from southern China, Southeast Asia, and northeast India and causes a life-threatening invasive mycosis—— Talaromycosis (Vanittanakom et al., 2006; Samson et al., 2011; Limper et al., 2017). With the increase in immunosuppressive therapy and the prevalence of AIDS, talaromycosis has become more common in clinical practice. Human infection probably occurs through inhalation of fungal spores from the contaminated surroundings (Vanittanakom et al., 2006), causing disseminated disease involving multiple organs/systems such as lungs, blood, bone marrow, lymphatics, and skin (Limper et al., 2017). In HIV-infected patients, *T. marneffei* is a common opportunistic pathogen after *Mycobacterium tuberculosis*, *Pneumocystis jirovecii*, and *Cryptococcus*, with a mortality of up to 30% (Hu et al., 2013; Jiang et al., 2019). Therefore, early and rapid diagnosis is crucial for antifungal treatment, improving the outcomes, and reducing mortality.

Clinical manifestations of talaromycosis in HIV-infected patients are complex, diverse, and non-specific. So, clinical diagnosis is mainly dependent on laboratory assays. Currently, the culture and species identification of clinical specimens is the golden standard for the diagnosis (Ning et al., 2018). However, it takes 10-14 days to yield a positive result, delaying the early diagnosis and timely treatment (Andrianopoulos, 2020; Chen et al., 2022). In addition, the culture results may be negative because of the low fungal loads in the early stage of infection, which will lead to a missed diagnosis. Microscope findings of yeast organizations in smear staining skin can make a presumptive diagnosis not feasible for patients without skin lesions (Limper et al., 2017; Chen et al., 2020). (1,3)-β-D-glucan (BDG) test and Galactomannan (GM) test were rapid and sensitive (Li et al., 2020), but both of them were unspecific and could not be used for definitive diagnosis. In recent years, novel nonculture-based assays like ELISA and PCR have been explored for early and rapid diagnosis. However, their sensitivity was 82% and 84%, respectively, which cannot meet the clinical diagnosis needs (Wang et al., 2011; Hien et al., 2016; Lu et al., 2016; Ning et al., 2018; Pruksaphon et al., 2018; Ly et al., 2020). *T. marneffei* is not a usually suspected infection pathogen in non-HIV-infected patients or immunocompromised patients (Li et al., 2022). Furthermore, the research based on PCR or ELISA is rarely carried out in these patients, limiting the early diagnostic performance of these two detection methods.

Metagenomic Next-Generation Sequencing (mNGS) is the application of Next-generation sequencing (NGS) technology in clinical microbiological testing. Compared with the traditional pathogen detection methods, mNGS was faster and unbiased, which can simultaneously detect various infectious pathogens, including viruses, bacteria, fungi, and parasites (Gu et al., 2019). Currently, mNGS has been applied in many infectious diseases, especially in unconventional pathogens, novel pathogens, and mixed infections (Duan et al., 2021). However, only few case reports have reported the usefulness of mNGS in the diagnosis of talaromycosis (Shi et al., 2021; Zhou et al., 2021). The performance of mNGS for the rapid diagnosis of talaromycosis in HIV patients remained unknown. Consequently, this study aimed to evaluate the diagnostic performance of this technique in HIV/AIDS individuals with *T. marneffei* infection.

## 2 Methods

### 2.1 Study design and subjects

This study retrospectively analyzed the HIV-infected patients hospitalized in the Department of Infection and Immunology of Changsha First Hospital (China) from January 2021 to March 2022. Patients who were suspected of opportunistic infection and underwent routine microbiological examination and mNGS in parallel were enrolled. The culture and serum GM results were collected within 7 days of admission. Patients meeting the following criteria were excluded: (1) The type of sample tested for mNGS was not cultured, (2) Culture and GM testing more than 7 days after admission, and (3) clinical data were incomplete.

Diagnosis of talaromycosis refers to the previous guidelines and expert consensus (AIDS-Associated Opportunistic Infections Research Group of the National Science and Technology Major Project of China During the 13th Five-Year Plan Period, 2020; AIDS and Hepatitis C Professional Group, 2021). The criteria were as follows:(1) with fever or respiratory/gastrointestinal abnormality, papulonecrotic skin lesions, lymphadenopathy, Splenomegaly, Hepatomegaly, anemia; (2) computed tomography suggested fungal infection; (3) exclude other fungal infections; (4) the talaromycosis-related symptoms and index resolved after anti-*T. marneffei* treatment; (5) *T. marneffei* was identified by culture or specific yeast found by microscopic examination from clinical samples. The clinical final diagnosis was made if (1) and (5) above can be met or if (1)-(4) above can be met. Non-talaromycosis refers to cases with etiology-proven other infectious diseases or non-infectious diseases. The clinical final diagnosis was used as a reference standard, and cases were classified into the TM group and the non-TM group.

### 2.2 Process of mNGS

#### 2.2.1 DNA extraction

BALF, blood, and bone marrow were collected according to standard operating procedures. 450μl of BALF or bone marrow from patients were mixed with 7.2μl lysozyme, holding 30℃ for 10 min. Then the mixture was transferred to a 2-ml microcentrifuge tube with 250μl 0.5-mm glass beads. The fastprep-24™ 5G instrument was used for vibration crushing, and reaction conditions were 45s*2cycles with an interval of 2 min. Then the supernatant was transferred to a 1.5-ml tube. Finally, a volume of 300μl supernatant or serum was extracted using the TIANamp Micro DNA Kit (TIANGEN BIOTECH) according to the operating manual.

#### 2.2.2 Construction of DNA library and sequencing

For DNA Library construction, the extracted DNA above was processed for breaking, end repair, adapter ligation, and PCR amplification using MGIEasy Cell-free DNA Library Prep Set (MGI Tech). DNA library was detected by Qubit 2.0 (Invitrogen) for quality control. The double-stranded liner DNA library was formed into single-stranded circular DNA by undergoing melting transition and circularization. Then, it was generated to DNA Nanoballs (DNB) through rolling circle amplification (RCA) technology. Qualified DNB was loaded on the chip and ran the “SE50+10” program on the MGISEQ-2000 or MGISEQ-50 platform (BGI Genomics) for sequencing.

#### 2.2.3 Bioinformatic analysis and criteria for positive results

High-quality data were obtained by removing low-quality bases, linker sequences, and short sequences (< 35 bp). The process of subtracting the human host sequence and aligning the remaining sequences were described in the previous study (Jin et al., 2020). The remaining data by removal of low-complexity reads were classified by simultaneously aligning to Pathogens metagenomics Database (PMDB). The PMDB contains the pathogen consisting of bacteria, fungi, viruses and parasites. The classification reference databases were downloaded from NCBI (ftp://ftp.ncbi.nlm.nih.gov/genomes/). RefSeq contains 4945 whole genome sequence of viral taxa, 6350 bacteral genomes or scaffolds, 1064 fungi related to human infection, and 234 parasites associated with human diseases. Positive criteria of mNGS results: A. A species-specific strictly mapped reads number (SMRN) per million ratio or SDSMRN of *T. marneffei* was≥1; B. Other pathogens were defined as the same in the previous study (Peng et al., 2021). The strictly mapped reads number means that, on the basis of meeting the conditions for mapped reads, and the proportion of mapped length in the statistical alignment results is greater than or equal to 90%. The base mismatch rate is less than or equal to 4% or the virus base mismatch rate is less than or equal to 8%, the optimal alignment score is greater than or equal to 30, and the sequence mapped frequency is 1.

### 2.3 Process of PCR

DNA was extracted according to the steps of 2. 2.1. The amplification was performed in 20μl reaction mix containing 10μl of 2 × TaqMan Universal Master Mix (Applied Biosystems, USA), 1μl (5μM) for each primer, 1μl (5μM) of the TaqMan probe, 5μl of template DNA and 2μl of RNase-free water. The conditions used were: 95℃ for 10 min and 45 cycles of 95℃ for 15 sec, 60℃ for 1 min, and annealing and extension at 60℃ for 1 min. PCR was performed using ABI 7500 (Applied Biosystems, USA), and controls and standard curve were performed in each run. The Cq value≤ 40 was determined as positive. More detailed of PCR method refer to the description of Li, etc. (Li et al., 2020).

### 2.4 Statistical analysis

The numerical variables were expressed as medians and interquartile ranges, and compared by the *Mann-Whitney U* test. The nominal variables were described by counts and percentages, and compared by the *chi-square* test. Sensitivity, specificity, positive predictive value (PPV), and negative predictive value (NPV) with 95% confidence intervals (CI) of mNGS in the diagnosis of talaromycosis were calculated. *Spearman’s* test was performed to analyze correlation. *P* values < 0.05 were considered significant.

### 2.5 Ethics Statement

The study was approved by the Ethics Committee of Changsha First Hospital, and carried out by relevant guidelines. Patients included in the study were informed and signed the consent for mNGS testing.

## 3 Results

### 3.1 Clinical characteristics and laboratory findings

A total of 208 patients were enrolled in this study, with 60 cases for the cases group (TM group) and 148 cases for the control group (non-TM group) according to the clinical final diagnosis. The clinical characteristics and laboratory findings were shown in **Table 1**. The majority of enrolled participants were male, and there was no difference in the sex composition ratio between the two groups (78.3% *VS* 85.5%, P = 0.186). However, the TM group had a younger median age (39 *VS* 51, P<0.001). The most common clinical symptoms in TM patients were fever (90.0%), lymphadenopathy (85.0%), respiratory symptoms (76.7%), and splenomegaly/hepatomegaly (43.3%). Unexpectedly, skin lesions (25.0%) ranked last. In addition to respiratory and gastrointestinal symptoms, the others had a higher incidence than non-TM patients (*P* < 0.05). Chest Computed tomography (CT) abnormalities in two groups shared a similar high frequency (100% *VS* 98.6%, *P* = 0.366).

**Table 1:** Clinical data of TM and non-TM patients.

Characteristic	TM	non-TM	*P*-value
Cases	60	148	
Age (years)	39 (30-49)	51 (39-59)	0
Sex (male)	47 (78.3)	127 (85.5)	0.186
Clinical manifestations			
Fever	54 (90.0)	87 (58.8)	0
Respiratory symptoms	46 (76.7)	116 (78.4)	0.788
Gastrointestinal symptoms	16 (26.7)	25 (16.9)	0.108
Skin lesions	15 (25.0)	20 (13.6)	0.045
Splenomegaly/Hepatomegaly	26 (43.3)	22 (14.9)	0
Lymphadenopathy	51 (85.0)	50 (33.8)	0
Chest CT abnormalities	60 (100.0)	146 (98.6)	0.336
Laboratory test			
white blood cell (×10^9^/L)	3.5 (2.2-5.1)	4.5 (3.2-6.7)	0.008
hemoglobin (g/L)	97 (86-106)	112 (96-128)	0
platelet (×10^9^/L)	113 (57-184)	185 (134-243)	0
procalcitonin (ng/ml)	0.49 (0.06-2.45)	0.06 (0.05-0.21)	0
C-reactive protein (mg/L)	59.6 (19.0-87.7)	29.4 (9.4-70.3)	0.014
ADA(U/L)	30.2 (19.5-47.0)	18.6 (12.7-28.3)	0
LDH(U/L)	297.4 (220.8-458.4)	242.4 (179.4-361.8)	0.003
Serum BDG (pg/ml)	87.9 (42.8-244.1)	42.4 (42.4-102.0)	0
CD4^+^ T cell (cells/ul)	19 (12-37)	60 (21-159)	0
≤50 cells/ul	52 (86.7)	66 (44.6)	0
50-200 cells/ul	8 (13.3)	51 (34.5)	–
>200 cells/ul	0 (0)	31 (20.9)	–

TM, talaromycosis; CT, computed tomography; ADA, adenosine deaminase; LDH, lactate dehydrogenase; BDG, (1;3)-β-D-glucan.

Significant differences in laboratory test results were observed between TM and non-TM group (*P* < 0.05). In the TM group, the indexes representing bone marrow hematopoietic function, such as hemoglobin, white blood cell, and platelet, were lower than those in the non-TM group. The non-specific inflammatory markers indicating infection, such as procalcitonin, C-reactive protein, adenosine deaminase (ADA), and lactate dehydrogenase (LDH), were significantly increased in the TM group. The median serum BDG test in TM patients was 87.9 pg/ml, remarkably higher than the non-TM patients (42.4 pg/ml). In the TM group, the median CD4^+^ T cell count was 19 cells/ul, significantly lower than the non-TM group (60 cells/ul). No patients had CD4^+^ T cell counts over 200 cells/ul, and 86.7% of patients had CD4^+^ T cell counts less than 50 cells/ul, which was significantly higher than the non-TM group (44.6%, *P* < 0.001).

### 3.2 Diagnostic performance and comparison of mNGS, culture, and GM in talaromycosis

In the TM group, mNGS of blood, bone marrow, and BALF were performed in 13 cases, 5 cases, and 42 cases, respectively. 5 bone marrow and 42 BALF samples were cultured in parallel. 4 patients had different type samples for mNGS testing, while it collected not in the same day. So, we just included the earliest collected sample types in this study. In the non-TM group, mNGS test were performed in 22 blood samples, 3 bone marrow samples, and 123 BALF samples, respectively. 3 bone marrow and 123 BALF samples were cultured in parallel. Blood cultures and serum GM were performed in all 208 cases. The test results are shown in **Table 2**.

**Table 2:** Diagnostic performance of mNGS, culture and Serum GM in TM and non-TM patients.

Test	Samples	TM		non-TM		Sensitivity	Specificity	PPV	NPV
		+	–	+	–	(95%CI)	(95%CI)	(95%CI)	(95%CI)
**mNGS**	Blood	13	0	0	22	100%	100%	100%	100%
						(71.7-100.0)	(81.5-100.0)	(71.7-100.0)	(81.5-100)
	BALF	41	1	2	121	97.6%	98.4%	95.3%	99.2%
						(85.9-99.9)	(93.7-99.7)	(82.9-99.2)	(94.9-100.0)
	Bone marrow	5	0	0	3	100%	100%	100%	100%
						(46.3-100)	(31.0-100)	(46.3-100)	(31.0-100)
	**Total**	**59**	**1**	**2**	**146**	98.3%	98.6%	96.7%	99.3%
						(89.8-99.9)	(94.7-99.7)	(87.6-99.4)	(95.7-99.9)
**Culture**	Blood	32	28	0	148	53.3%	100%	100%	84.1%
						(40.1-66.1)	(96.8-100.0)	(86.7-100.0)	(77.7-89.0)
	BALF	12	30	0	123	28.6%	100%	100%	80.4%
						(16.2-44.8)	(96.2-100.0)	(69.9-100.0)	(73.0-86.2)
	Bone marrow	4	1	0	3	80.0%	100%	100%	75.0%
						(30.0-98.9)	(31.0-100.0)	(39.6-100.0)	(21.9-98.7)
	**Total^#^**	**40**	**20**	**0**	**148**	66.7%^##^	100%	100%	88.0%^##^
						(53.2-77.9)	(96.8-100.0)	(89.0-100.0)	(81.9-92.4)
**Serum GM**	Total^*^	**50**	**10**	**12**	**136**	83.3%^**^	91.9%^**^	80.6%^**^	93.1%^**^
						(71.0-91.2)	(85.9-95.5)	(68.3-89.2)	(87.4-96.5)

GM, galactomannan; CI, confidence intervals; PPV, positive predictive value; NPV, negative predictive value. ^#^ culture positive was defined as confirmed T. marneffei in any specimen of blood, bone marrow and BALF, ^*^ Serum GM ≥0.5 was defined as positive, ^##^ P<0.001 when comparing mNGS total with culture total, ^**^ P<0.05 when comparing mNGS total with Serum GM total.

In current clinical practice, culture was a routine method for definitive diagnosis of talaromycosis, and serum GM test was widely used in the rapid adjuvant diagnosis of talaromycosis. Our study defined the optical density value of serum GM ≥ 0.5 as positive. And culture positive was defined as confirmed *T. marneffei* in any specimen of blood, bone marrow and BALF. The sensitivity of mNGS test reached 98.3% (95% CI,89.8-99.9), which was significantly higher than culture (66.7% [95% CI,53.2-77.9], *P* < 0.001) and serum GM (83.3% [95% CI,71.0-91.2], *P* < 0.05). The specificity of mNGS (98.6% [95% CI,94.7-99.7]) was superior to serum GM (91.9% [95% CI,85.9-95.5], *P* < 0.05), and similar to culture (100.0% [95% CI,96.8-100.0], *P* = 0.156). Meanwhile, mNGS also had excellent positive predict value (PPV) and negative predict value (NPV) of 96.7% (95% CI,87.6-99.4) and 99.3% (95% CI,95.7-99.9), respectively, and both significantly exceed serum GM test (*P* < 0.05). More detailed diagnostic performance indices were listed in **Table 2**.

**Figure 1:**
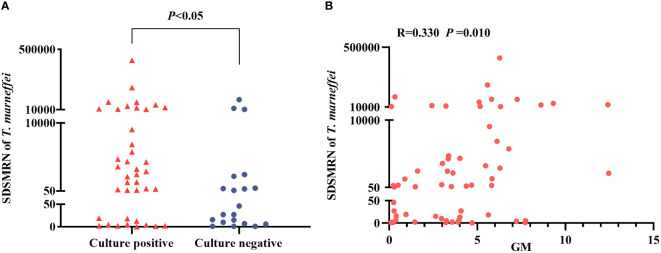
Analysis of the strictly mapped reads number per million ratio (SDSMRN) of *T. marneffei* detected by metagenomic Next-Generation Sequencing(mNGS). **(A)** Comparison of the SDSMRN of *T. marneffei* between culture positive and culture negative cases (*Mann-Whitney U* test). **(B)** Correlation analysis between the SDSMRN of *T. marneffei* and serum galactomannan (GM) levels (*Spearman’s* test). *P<0.05* statistic difference.

The SDSMRN of *T. marneffei* detected by mNGS in the culture-positive patients was significantly higher than in the culture-negative patients (*P* < 0.05) (**Figure 1A**). Moreover, *Spearman’s* analysis shows a positive correlation between the SDSMRN of *T. marneffei* and serum GM levels in TM patients (R = 0.330, *P* = 0.010) (**Figure 1B**).

### 3.3 PCR validation of *T. marnefei* detected by mNGS

Among the 208 cases, *T. marnefei* were detected by mNGS in 61 cases, including 59 in the TM group and 2 in the non-TM group. 39 out of 59 had been confirmed by culture. The remaining 22 cases were verified by real-time polymerase chain reaction (PCR) (Li et al., 2020). 2 cases in the non-TM group were negative. In the TM group, 3 cases (1 BALF, 1 blood and 1 bone marrow) were negative. 16 BALF samples and 1 blood sample detected *T. marnefei* by the specific PCR.

### 3.4 Positive rates of mNGS in different types of samples

**Figure 2:**
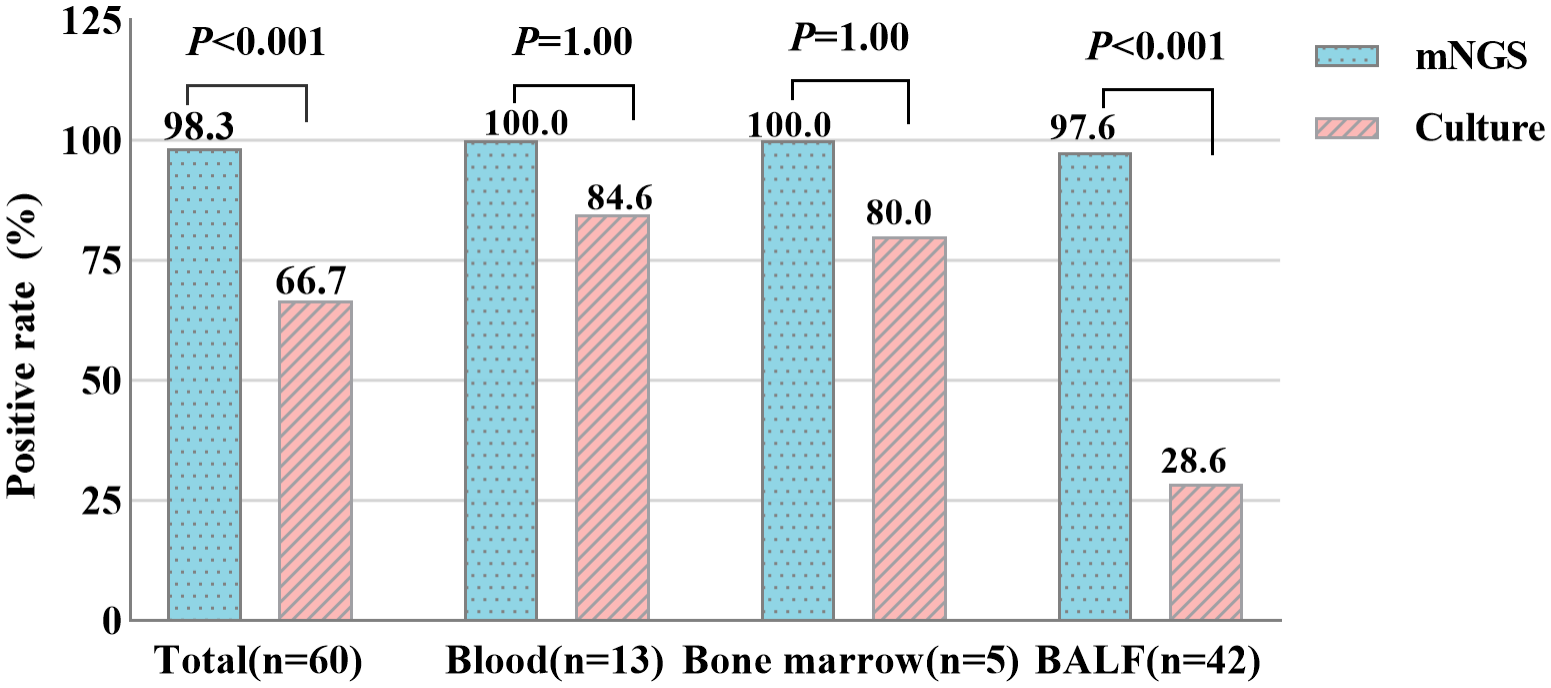
Positive rate comparison of metagenomic Next-Generation Sequencing (mNGS) and culture in blood, bone marrow and bronchoalveolar lavage fluid (BALF) samples (*chi-square* test). *P<0.05* statistic difference.

In the TM group, 12 of the 13 blood samples were both positive for mNGS and culture assay, while one sample was positive for mNGS only. The positive rates of mNGS and culture in blood samples were 100.0% and 84.6%, respectively, with no difference (*P* > 0.05). 1 of 5 bone marrow samples tested positive for mNGS only, the remaining samples were positive for the two assays, and the positive rates were 100.0% and 80.0%, respectively. However, for all 42 BALF samples, only 12 were positive for both methods, 1 was negative for mNGS, and as many as 29 were positive for mNGS only. The positive rates of these two methods in BALF samples were significantly different, and mNGS was significantly higher than culture (97.6% VS 28.6%, *P* < 0.001) (**Figure 2**).

### 3.5 Analysis of concurrent pathogens detected by mNGS

**Figure 3:**
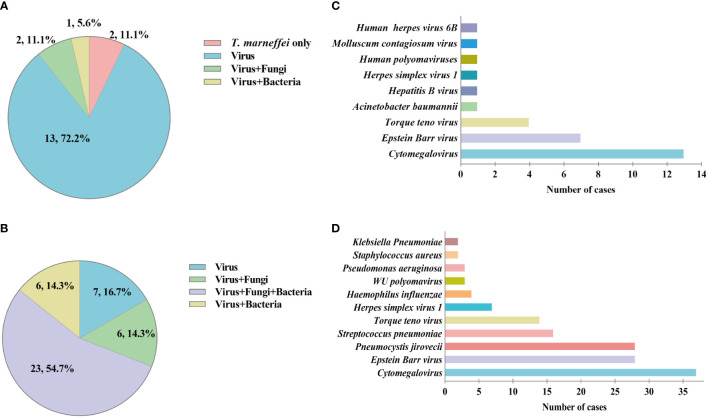
Mixed infection and concurrent pathogens detected by metagenomic Next-Generation Sequencing (mNGS) in talaromycosis (TM) patients. **(A)** Proportion of different types of mixed infection in blood and bone marrow samples. **(B)** Proportion of different types of mixed infection in bronchoalveolar lavage fluid (BALF) samples. **(C)** Numbers of various concurrent pathogens in blood and bone marrow samples. **(D)** Numbers of various concurrent pathogens in BALF samples.

In the TM group, 58 cases (96.7%) of putative co-infections were identified based on mNGS. It should be noted that, due to the limitation of the DNA detection procedure, only DNA viruses were detected in this study. The types of co-infection pathogens identified by mNGS in different kinds of specimens were different. Among 18 blood or bone marrow samples, *T. marneffei*-virus was the most common mixed infection (72.2%) (**Figure 3A**). While, in 42 BALF samples, *T. marneffei*-virus-fungi-bacteria mixed infection accounted for the majority (54.7%) (**Figure 3B**). *Cytomegalovirus* and *Epstein-Barr virus* were the top two common concurrent pathogens (**Figures 3C, D**). Particularly, in BALF samples, mNGS detected 28 cases of *Pneumocystis jirovecii*, a common opportunistic infection pathogen in HIV-infected patients (**Figure 3D**). In addition, mNGS also detected one case of *Cryptococcus neoformans*, *Mycobacterium tuberculosis*, and *Mycobacterium avium*.

### 3.6 Adjustments of antimicrobial therapy after mNGS

By searching electronic medical records, we collected the adjustments of antimicrobial treatment after mNGS testing in 60 TM patients. A total of 36 cases (60.0%) were adjusted for antimicrobial therapy based on mNGS results. 24 cases (40.0%) were added with amphotericin B(AMB), and 3 (5.0%) cases were added with itraconazole and started anti-*T. marneffei* treatment, respectively. 3 (5.0%) cases were added with trimethoprim-sulfamethoxazole (TMP-SMZ), and 8(13.3%) cases were added with antiviral drugs. All the adjustments were shown in **Table 3**. BALF (28/42, 66.7%) sample was principal for adjustments of antimicrobial therapy. We mainly observed and recorded the effect of adding AMB and Itraconazole for anti- *T. marneffei* treatment, and we found that the patient’s clinical symptoms improved, blood culture turned negative 1-2 weeks later, and chest CT improved 2 weeks later.

**Table 3:** Adjustments of antimicrobial therapy after mNGS on TM patients.

Adjustments	Cases (n [%])
Total adjustments	36 (60.0%)
Add AMB	24 (40.0)
Add Itraconazole	3 (5.0%)
Add TMP-SMZ	3 (5.0%)
Add antiviral drugs	8 (13.3%)
Add antibacterial drugs	1 (1.6%)

AMB, amphotericin B; TMP-SMZ, trimethoprim-sulfamethoxazole.

## 4 Discussions

This study retrospectively analyzed the results of using the mNGS method to detect *T. marneffei* in different samples of HIV patients and found that mNGS showed high sensitivity and specificity of 98.3% and 98.6%, respectively. Moreover, we found that mNGS demonstrated apparent advantages in detecting BALF samples and mixed infection.

At present, the culture of clinic samples was the golden standard to identify *T. marneffei* infection. The statistical results of this study showed that the median turnaround time (TAT) of positive culture was 7 (6-9) days, with a sensitivity of 66.7%. Time-consuming and insensitive lead to the missed and delayed diagnosis of clinical application, which was associated with high mortality (Hu et al., 2013; Qiu et al., 2019). Serum GM detection has been applied to detecting Aspergillus and *T. marneffei*, which cannot identify a specific genus. The sensitivity of the serum GM assay in this experiment was 83.3%, which was similar to that of Li et al. (Li et al., 2020), but was lower than that of Zheng et al (Zheng et al., 2015). This may be caused by the latter using 1.0 as an optical density cutoff value.

mNGS is an emerging molecular biology-based pathogen detection method with the advantages of rapidity, sensitivity and specificity. In our experiment, the median TAT of mNGS was 1 (1-2) days, which was shorter than culture. Comparing the three detection methods, we found that the sensitivity of mNGS was significantly higher than culture (98.3% vs 66.7%, P<0.001) and serum GM (98.3% vs 83.3%, P<0.05), and the specificity of mNGS was similar to culture. The detection assay based on PCR has not been widely used in the clinic, and a commercial PCR kit for detecting *T. marneffei* is not available in China at present. In our study, we used the PCR method established by Li et al. to verify 22 mNGS positive specimens (Li et al., 2020). 3 cases of clinically confirmed talaromycosis were not detected by PCR, which may be related to the sensitivity or long-term preservation of samples. Because we only performed PCR on a small number of samples, we did not compare the diagnostic performance of PCR and mNGS in talaromycosis. Recent studies show that the PCR-based method has a sensitivity of 70.4-91.0% (Hien et al., 2016; Lu et al., 2016; Li et al., 2020), which is lower than mNGS in this study. We speculate that the loss of nucleic acid during extraction may have more impacts on the detecting of specific fragments in PCR. The specificity of PCR-based detection methods varies greatly, ranging from 63.0 to 100.0% (Hien et al., 2016; Lu et al., 2016; Li et al., 2020). *MP1P* antigen of *T. marneffei* was developed for rapid detection and showed a sensitivity of only 86.3% (n=372) and 72.0% (n=93), respectively, in two studies of relatively large sample size (Thu et al., 2021; Chen et al., 2022). Compared with the detection methods mentioned above, mNGS demonstrates better sensitivity and specificity in the rapid diagnosis of talaromycosis in HIV patients.

Talaromycosis is a systemic fungal infection that can spread along the reticuloendothelial system. So, selecting appropriate samples is critical to improving the positive rate and rapid diagnosis. Microscopic examination of scraping pieces of specific skin lesions in the central depression is the most rapid and straightforward method for a preliminary diagnosis. However, in this study, 75% of patients in the TM group had no skin lesions, which could not be identified by this method. Cultures of blood and bone marrow samples have been recommended and well-studied, but positive only during late-stage infections, and 30-50% of infected individuals are missed (Thu et al., 2021). There was no significant difference in the positive rate of mNGS and culture in blood and bone marrow samples in our study, demonstrates that mNGS was not advantageous with blood and bone marrow.The lung is the primary entry portal and also has the highest burden of talaromycosis (Narayanasamy et al., 2021). However, BALF appears to be a less studied sample type. A retrospective analysis of a large sampling showed that the positive rate of BALF cultures was only 28.1% (n = 427) (Ying et al., 2020), which was similar to the results of our study. Surprisingly, the positive rate of mNGS in BALF reachs 97.6%, significantly higher than in culture (97.6% VS 28.6%, P < 0.001). Moreover, our study also found that the SDSMRN of *T. marneffei* in culture-negative patients was significantly lower than in culture-positive patients. Given this, we speculate that this is due to the sensitivity limitation, and the early low fungal load infection in the pulmonary cannot be detected by culture in BALF samples. Furthermore, the isolation culture of *T. marneffei* from BALF samples in advanced HIV/AIDS patients is challenging due to mixed pulmonary infection with multiple pathogens. As a result, pulmonary talaromycosis may have been neglected and underestimated in current practice and reports. These results demonstrate the apparent advantages of mNGS in BALF samples, which may greatly facilitate the early detection of *T. marneffei* in HIV-infected individuals with manifestations infection.

The pathogen spectrum of mNGS is broad, and it has significant advantages in identifying mixed infections. In advanced HIV/AIDS patients with CD4^+^T cell counts of less than 200 cells/ul, the risk of infection with *T. marneffei* was significantly increased, the same as other pathogens (such as *Mycobacterium tuberculosis*, *Cryptococcus*, and *Pneumocystis jirovecii*) (Jiang et al., 2019; Qin et al., 2020), and the risk of mixed infections was also increased. The application of mNGS in this population may give full play to its cost, which is confirmed by our research. Based on the routine microbiological examination, 42.4% talaromycosis patients were identified with other opportunistic infections (Ying et al., 2020), but it was up to 96.7% by mNGS, which is significantly higher. Aside from bacteria, multiple types of pathogens were detected by mNGS, especially for viruses (such as *Cytomegalovirus* and *Epstein-Barr virus*) and fungi. Advanced HIV/AIDS patients with a high risk of opportunistic infections can significantly benefit from mNGS testing. Most notably, in BALF samples, we observed another common unculturable opportunistic infection pathogen, *Pneumocystis jirovecii*, with several cases ranking third, implying that BALF samples could be a good option for effectively identifying the mixed infections of *T. marneffei* and *Pneumocystis jirovecii.* In our observation, 60.0% of cases had an adjusted antimicrobial regimen, and 45% of cases started anti-*T. marneffei* based on mNGS results. It suggested that the powerful technology may guide the safe and effective use of antimicrobial drugs in the future.

Despite the significant advantages of mNGS, there are still some challenges in clinical application. How to scientifically interpret mNGS results remains problem which cannot be ignored. In agreement with previous study, *T. marneffei* was considered as an exogenous pathogenic fungus, not colonized in the pulmonary (Limper et al., 2017; Chen et al., 2021). So, the SDSMRN of *T. marneffei* ≥1 was considered positive in our study. However, the microbes and nucleic acid contamination from the process of sample collection and experiments may also be detected with low mNGS reads. Therefore, the mNGS result must be carefully analyzed based on a comprehensive analysis of clinical manifestations and other laboratory tests. Presently, few studies have reported the distinction of pathogens detected by mNGS among contamination, colonization and infection. Authoritative normative guideline was urgently needed. In addition, the high cost of mNGS limits widespread promotion, so the type of patients who can benefit more from this technology should be carefully considered by clinicians.

Our study has some limitations. Firstly, this is a single-centre retrospective analysis. Secondly, this study uses clinical final diagnosis as the classification standard, which is prone to classification bias. Thirdly, this study did not analyze the effect of prophylactic or therapeutic antifungals on mNGS outcomes. In future practice, we should pay more attention to pulmonary talaromycosis. Non-HIV individuals should be included to assess the diagnostic performance of mNGS in talaromycosis, and multicenter prospective studies are also necessary.

In conclusion, mNGS is a powerful technique with high specificity and sensitivity for the rapid diagnosis of talaromycosis. mNGS of BALF samples may be a good option for early identification of *T. marneffei* in HIV-infected individuals with manifestations of infection. Moreover, mNGS shows excellent performance in mixed infection, which benefits timely treatment and potential mortality reduction in HIV-infected patients.

## Data availability statement

The datasets presented in this study are deposited in online repositories. The names of the repository/repositories and accession number(s) can be found below: http://db.cngb.org/,CNP0003573.

## Ethics statement

The study was performed following with the Ethics Committee of Changsha First Hospital, the Helsinki Declaration of 1964 and its later amendments. Patients included in this study were informed and signed the consent for mNGS testing.

## Author contributions

YM, HS, YC, JH and WH designed the study. YM, CY and JL collected and sorted out the original data. YM and WH analyzed the data. QJ participated in the production of figures and tables. YM drafted the manuscript of the paper. WH revised the manuscript. All authors contributed to the article and approved the submitted version.

## Acknowledgments

We acknowledge the contributions of authors and patients who participated in this study. We are particularly grateful to the professor of CunWei Cao (Department of Dermatology and Venereology, the First Affiliated Hospital of Guangxi Medical University, Nanning, China) for providing PCR technical support and Yali Wang for English language editing.

## Conflict of interest

The authors declare that the research was conducted in the absence of any commercial or financial relationships that could be construed as a potential conflict of interest.

## Publisher’s note

All claims expressed in this article are solely those of the authors and do not necessarily represent those of their affiliated organizations, or those of the publisher, the editors and the reviewers. Any product that may be evaluated in this article, or claim that may be made by its manufacturer, is not guaranteed or endorsed by the publisher.
